# Causal relationship between body mass index and anal fistula: a two-sample Mendelian randomization study

**DOI:** 10.3389/fgene.2024.1406231

**Published:** 2024-07-25

**Authors:** Bo Chen, Yicheng Liu, Yueting Wang, Qingming Wang

**Affiliations:** ^1^ Department of Anorectal Surgery, Baoshan District Integrated Traditional Chinese and Western Medicine Hospital, Shanghai, China; ^2^ Department of Traditional Chinese Medicine, Juquan Xincheng Community Health Service Center, Shanghai, China; ^3^ Department of Anorectal Surgery, Shuguang Hospital Affiliated to Shanghai University of Traditional Chinese Medicine, Shanghai, China

**Keywords:** two sample Mendelian randomization, body mass index, BMI, anal fistula, causal relationship, incidence risk

## Abstract

**Background:**

Significant evidence has been documented regarding the intricate connection between the development of anal fistula (AF) and the composition of Body Mass Index (BMI). Nevertheless, due to the inherent limitations of reverse causality and confounders inherent in observational studies, this relationship remains unclarified. Our study aims to reveal the causal impact between BMI and AF, as well as identify its associated risk factors, thereby providing a more comprehensive understanding of this complex interaction.

**Methods:**

Single nucleotide polymorphisms (SNPs) identified through genome-wide association study (GWAS) databases were used as instrumental variables for analysis. BMI served as the exposure variable, with six pooled GWAS datasets included. AF was the outcome variable. The Inverse Variance Weighted (IVW) method was used as the primary analytical technique, with MR-Egger regression, Weighted Median (WME) estimation, and Multiplicity Residual Sum and Outlier (MR-PRESSO) tests serving as secondary validations of the IVW results. Odds ratios (OR) were utilized as indicators to evaluate the causal relationship between BMI and AF.

**Results:**

A total of 738 SNPs strongly associated with the exposure were identified as instrumental variables. The IVW results demonstrated a positive correlation between BMI and the risk of AF. The MR-Egger analysis yielded *p*-values greater than 0.05, indicating no pleiotropic effects among the selected SNPs. Cochran’s Q test also resulted in *p*-values greater than 0.05, suggesting no significant heterogeneity among the instrumental variables. The MR-PRESSO analysis revealed no horizontal pleiotropy or outliers potentially violating the causal assumption (*p* > 0.05).

**Conclusion:**

High BMI is positively associated with the risk of AF, and correcting BMI levels may have a preventive effect on the incidence of AF.

## Introduction

Anal fistula (AF) is primarily characterized by persistent or intermittent purulent, bloody, or mucinous secretions at the external orifice of the fistula, along with local symptoms of redness, swelling, heat, and pain. The severity of symptoms can vary significantly, often necessitating surgical intervention (Limura and Giordano, 2015; Hwang, 2022). Obesity, a slow-paced metabolic disease caused by multiple factors, refers to a pathological state of excessive accumulation of body fat, particularly triglycerides, resulting in a certain degree of overweight and excessive fat layer thickness (Pigeyre et al., 2016). A diagnosis of obesity is typically made when the body mass index (BMI) is ≥ 30 kg/m2. Epidemiological studies have shown that the prevalence of AF is 12.3 cases per 100,000 men and 5.6 cases per 100,000 women (Sainio, 1984). Over the past 40 years, the prevalence of obesity has increased from 3.2% to 10.8% among young and middle-aged men, and from 6.4% to 14.9% among women, while AF commonly occurs in individuals aged 20–40 years (NCD Risk Factor Collaboration NCD-RisC, 2016). Previous studies have found that obesity can promote inflammatory responses, metabolic dysfunction, and cancer risks (Iyengar et al., 2016; Kawai et al., 2021). Additionally, obesity will lead abnormalities in cell-mediated immune and phagocytic functions, resulting in anal fistula (Liu et al., 2011). Another meta-analysis found that an elevated BMI in patients with AF may be a potential factor for the high recurrence rate after AF surgery (Mei et al., 2019).

Currently, multiple studies suggest a positive correlation between BMI and the risk of AF occurrence. However, previous observational studies have been subject to biases such as confounding factors and reverse causality. Mendelian randomization (MR) is an epidemiological research method that can infer potential causal relationships and has been widely used in recent years for causality research in genome-wide association study (GWAS) data (Smith and Ebrahim, 2003; Evans and Davey Smith, 2015). This method uses single nucleotide polymorphisms (SNPs) as instrumental variables to infer the causal relationship between exposure factors and outcomes (Wang, 2020). Since the alleles of specific SNPs are randomly assigned at conception, and genetic variations precede the development of diseases, genetic variations are not influenced by potential confounding factors, thus avoiding the possibility of reverse causality. The aim of this study is to explore the causal impact of obesity on the risk of AF occurrence using a two-sample Mendelian randomization analysis approach.

## Materials and methods

### Analysis methods and data sources

In this study, BMI was treated as the exposure factor, and SNPs significantly associated with BMI were used as Instrumental Variables (IVs), with AF as the outcome variable. After eliminating outliers, the causal analysis was performed by using two sample MR analysis method, followed by heterogeneity test and pleiotropy test, and finally the reliability of the results was tested. IVs in MR study should meet three core assumptions (Greenland, 2000; Lawlor et al., 2008; Burgess et al., 2013): (1) The IVs are strongly correlated with the exposure factor; (2) The IVs affect the outcome only through the exposure factor and are not directly or indirectly related to the outcome through other pathways; (3) The IVs are unrelated to confounding factors in the “exposure-outcome” relationship. All data on the association between exposure factor variables BMI and outcome variable AF were sourced from the IEU Open GWAS project database. SNPs significantly associated with BMI were screened as IVs based on GWAS, and different MR methods were used to analyze the causal relationship between BMI and AF. The data population consisted of Europeans and did not include gender stratification. By reading the original literature, no sample overlap problems between the two groups of data were found. Ethical review and informed consent had been obtained in all original studies.

### Selection of IVs

**FIGURE 1:**
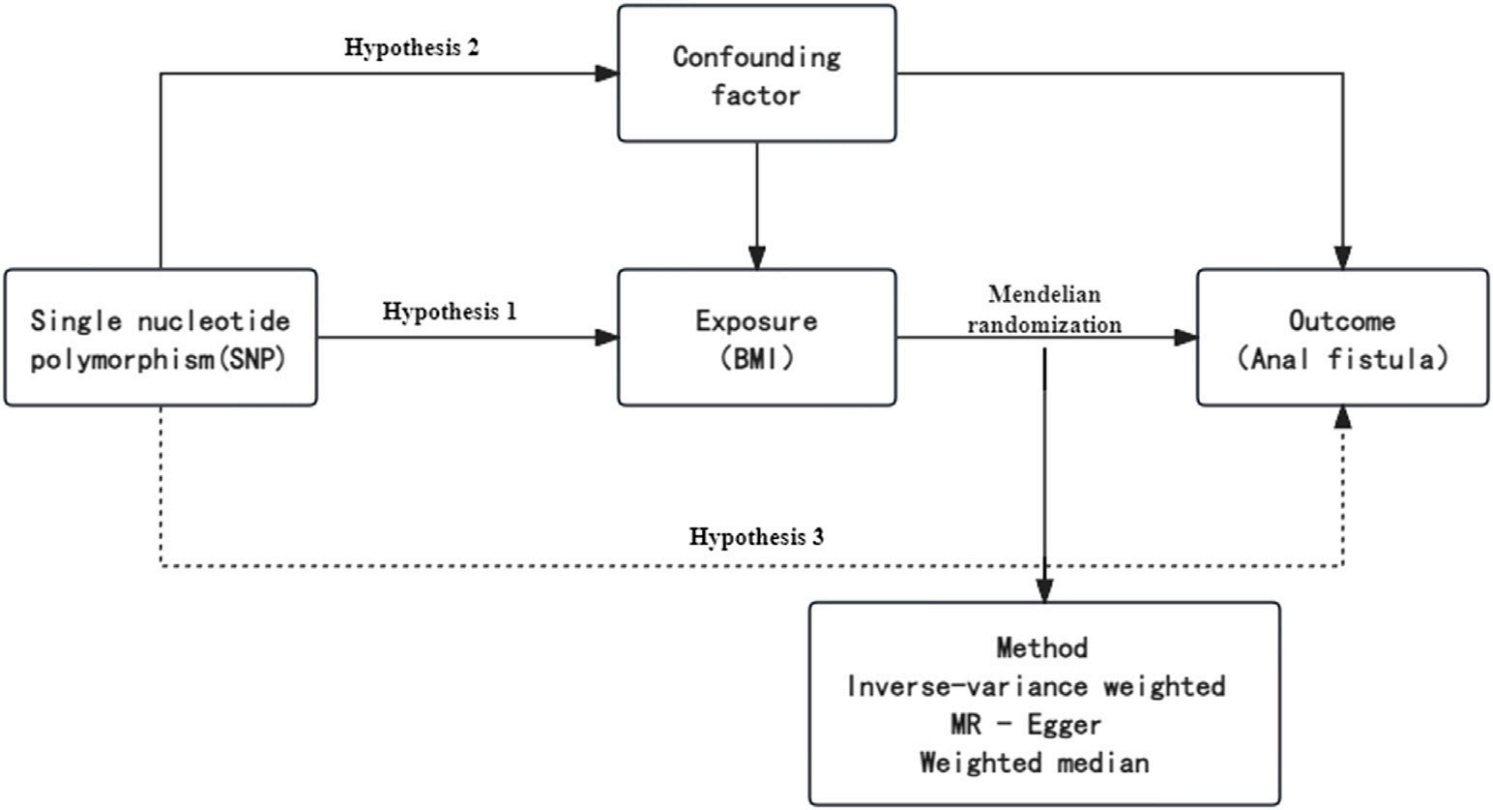
Schematic diagram of two sample Mendelian randomization models.

To avoid bias in the analysis that may be introduced by strong linkage disequilibrium (LD) relationships among SNPs, this study selected SNPs that were independent of each other and had a genome-wide association with AF from the database, with selection criteria including (Noyce et al., 2017): (1) Selecting IVs highly correlated with the exposure factor (BMI) as SNP sites (*p* < 5 × 10^-8); (2) Physical distance between genes >10,000 kb, and maintaining an r^2 value of <0.001 for LD between retained SNPs; (3) Removing SNPs with a minor allele frequency (MAF) ≤0.01; (4) Ensuring that the effect of SNPs on the exposure corresponds to the same allele as their effect on the outcome. A combined dataset of exposure and outcome was merged, containing the above IVs and their relationship with both the outcome and exposure factors, and palindromic SNPs were deleted. The remaining SNPs were the final IVs representing the exposure. The Weighted Median Estimate (WME) is a weighted empirical distribution function of ratio estimate values for all SNPs within the research range, which can reduce bias in the estimation of causal effects. A flowchart briefly presents the whole procedure in Figure 1.

### Two-sample MR analysis

This study used 3 MR methods to analyze the causal estimate of BMI on AF, mainly using Inverse Variance Weighted (IVW) analysis (Liu et al., 2011; Burgess et al., 2013), supplemented by WME (Bowden et al., 2015)^,^ and MR-Egger regression (Bowden et al., 2016). The IVW method was used as the main analysis method to assess the causal effect of BMI on AF. The IVW method combines the Wald ratio estimates of each IV in a meta-analysis into a weighted linear regression model for the association between the instrument and the result. When there is no pleiotropy of IVs, the IVW method has higher test efficiency and precision and can obtain the smallest effect estimate with minimal bias. Further, the intercept term of the MR-Egger regression was used to test whether there is genetic pleiotropy in the BMI-related IVs with AF, with *P* > 0.05 indicating that there is no pleiotropy between the instrumental and the outcome variables, and rejecting the null hypothesis indicates the presence of pleiotropy (Burgess and Thompson, 2017). The Cochran’s Q test was used to test the heterogeneity of the IVs, with *P* < 0.05 indicating heterogeneity (Burgess et al., 2016). The MR Pleiotropy Residual Sum and Outlier (MR-PRESSO) test was used to exclude outliers (Verbanck et al., 2018). A leave-one-out sensitivity analysis method was used to perform sensitivity analysis, examining the impact of each SNP on the results (Skrivankova et al., 2021). Stability of the results was checked by observing asymmetry in the funnel plot. This study conducted statistical analysis using the TwoSampleMR and MR-PRESSO software packages in R (version 4.3.1), with a significance level of α = 0.05.

## Results

### Selection of IVs

According to the instrumental variable screening standards, six highly relevant BMI exposure datasets with AF were selected from GWAS, and after excluding linkage disequilibrium, 738 SNPs from the GWAS database were used as IVs for two-sample Mendelian randomization analysis. Among them, six groups of BMI IVs contained 2,147,205 participants. The genetic correlation data of AF came from a GWAS of 463,010 participants in the United Kingdom Biobank, including 1,003 cases of AF. The population sources of both groups of data were European and included both males and females. Specific information about GWAS can be found in Table 1.

**TABLE 1:** Preliminary description on GWAS studies of BMI and AF.

GWAS ID	Exposure	PMID	Sample size	Total SNPs	Related SNPs	Population
ieu-a-94	BMI	23754948	60,586	2 736 876	3	European
ieu-a-785	BMI	25673413	152,893	2 477 659	14	European
ieu-b-40	BMI	30124842	681,275	2 336 260	218	European
ukb-a-248	BMI	-	336,107	10,894 596	131	European
ukb-b-2303	BMI	-	454,884	9 851 867	187	European
ukb-b-19953	BMI	-	461,460	9 851 867	185	European

### Causal effect analysis results of BMI and AF

**FIGURE 2:**
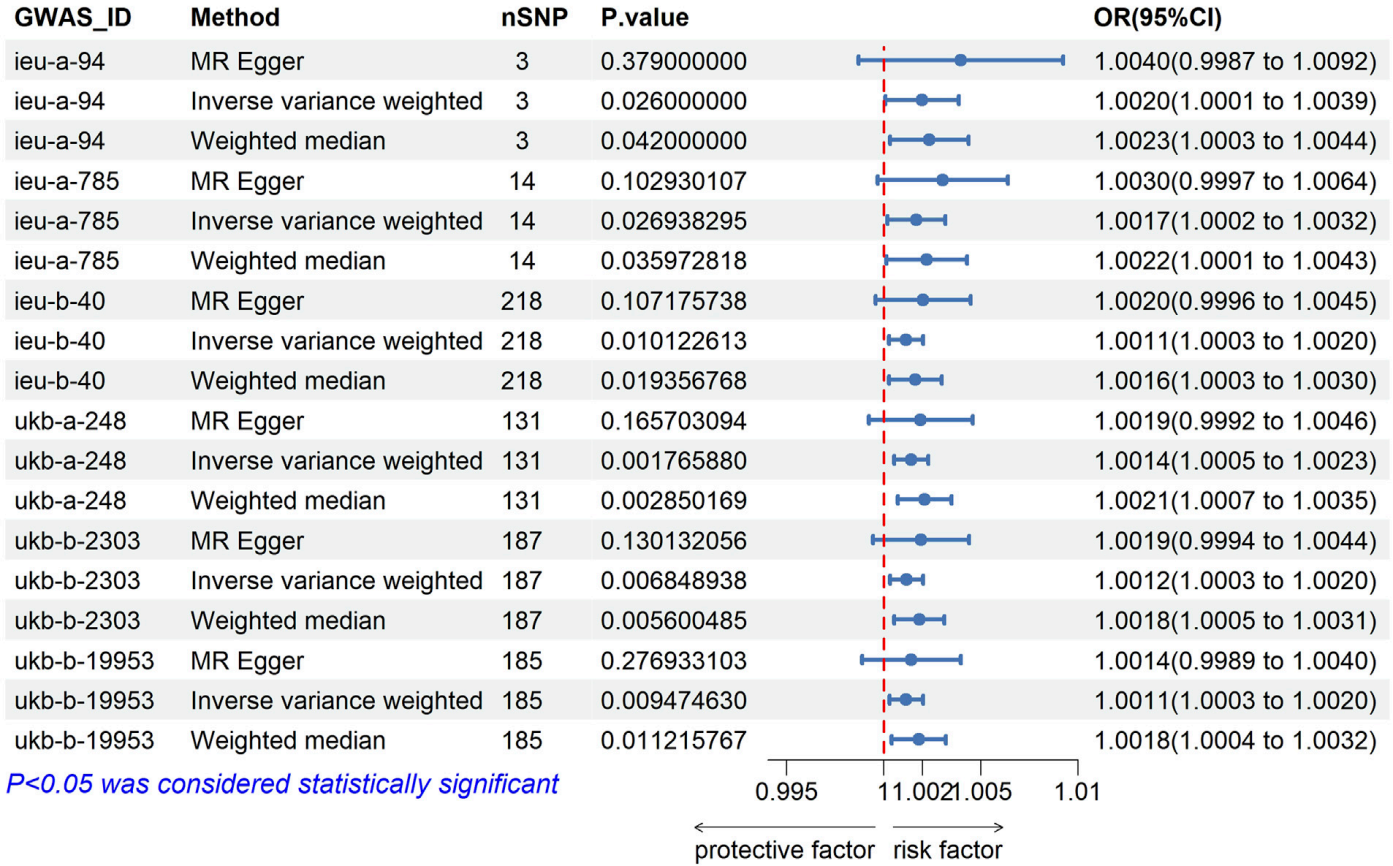
Forest plot of six groups of GWAS and AF.

**FIGURE 3:**
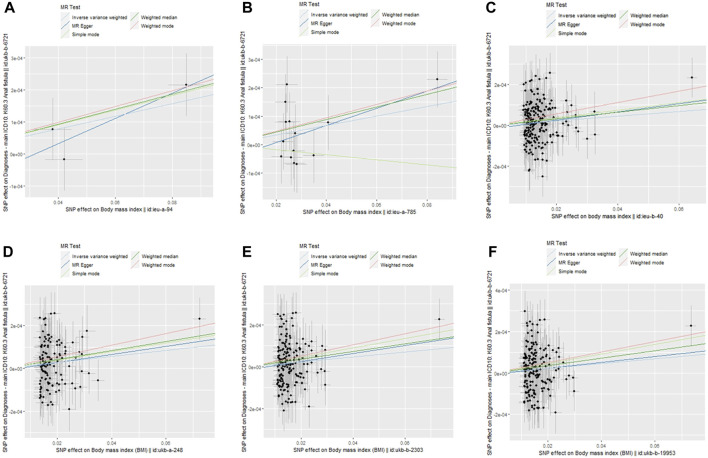
Scatter plots of the 5 MR tests in six causal associations from BMI features to anal fistula. SNP effects were plotted into lines for the inverse-variance weighted test (light blue line), weighted median estimator (green line), MR-Egger regression (blue line), weight median estimator (red line) and simple mode estimator (light green line). The slope of the line corresponded to the causal estimation.

After analysis, the F values of all six groups of BMI-related IVs were greater than 10, suggesting that the influencing factors of BMI were related to the risk of developing AF. The IVW method showed that the increase in genetic predicted BMI IVs ieug-a-94 (OR = 1.002, 95%*CI* 1.000–1.004, *p* = 0.026), ieug-a-785 (OR = 1.0017, 95%*CI* 1.001–1.004, *p* = 0.027), ieub-40 (OR = 1.001, 95%*CI* 1.000–1.003, *p* = 0.010), ukb-a-248 (OR = 1.001, 95%*CI* 1.001–1.002, *p* = 0.002), ukb-b-2303 (OR = 1.001, 95%*CI* 1.000–1.002, *p* = 0.007), and ukb-b-19953 (OR = 1.001, 95%*CI* 1.000–1.002, *p* = 0.009) was associated with an increased risk of AF, see Figures 1, 2. Table 2 lists the IVW results of some SNPs, OR values, and 95%CI. The β values of MR-egger regression and WME showed the same direction as the β values of the IVW method, indicating that the direction of causal effects obtained by the three methods was consistent (Table 2; Figures 2, 3).

**TABLE 2:** SNPs associated with BMI and AF.

GWAS ID	SNP	IVW					MR-egger		MR-PRESSO
		Se	*P*-Value	*Β* Value	*OR*	95%*CI*	*P*-Value	*Β* Value	Global test *P*-value
ieu-b-40	rs1296328	0.005	0.009	0.014	1.014	(1.004, 1.025)	0.107	0.002	0.607
	rs2479958	0.006	0.010	−0.016	0.984	(0.987,0.996)			
	rs8047395	0.0004	0.016	0.004	1.001	(1.000, 1.007)			
	rs2861683	0.007	0.020	0.016	1.004	(1.002,1.030)			
	rs2065418	0.006	0.020	0.014	1.014	(1.002,1.026)			
	rs10478110	0.010	0.033	0.021	1.021	(1.002, 1.041)			
ieu-a-94	rs13130484	0.003	0.432	0.002	1.002	(0.997,1.007)	0.379	0.004	-
	rs3101336	0.002	0.867	−0.0003	0.9996	(0.995, 1.004)			
	rs7193144	0.001	0.029	0.003	1.003	(1.000, 1.005)			
ieu-a-785	rs11604680	0.004	0.697	0.001	1.001	(0.994, 1.009)	0.103	0.003	0.607
	rs11676272	0.004	0.631	−0.002	0.998	(0.991, 1.006)			
	rs1222069	0.004	0.035	0.009	1.009	(1.001, 1.017)			
	rs12286929	0.004	0.665	−0.002	0.998	(0.990, 1.007)			
	rs13130484	0.002	0.432	0.002	1.002	(0.998, 1.007)			
	rs1421085	0.001	0.020	0.003	1.003	(1.000, 1.005)			
ukb-a-248	rs10100245	0.005	0.286	−0.005	0.995	(0.986, 1.004)	0.166	0.002	0.428
	rs10144067	0.005	0.599	0.003	1.003	(0.993, 1.012)			
	rs10187101	0.006	0.750	0.002	1.002	(0.990, 1.015)			
	rs10404726	0.005	0.472	−0.004	0.996	(0.987, 1.006)			
	rs10465231	0.006	0.150	0.008	1.008	(0.997, 1.020)			
	rs1064213	0.006	0.300	−0.006	0.994	(0.982,1.006)			
ukb-b-2303	rs10099330	0.008	0.494	−0.006	0.994	(0.978,1.011)	0.130	0.002	0.385
	rs10144067	0.005	0.599	0.003	1.003	(0.992,1.013)			
	rs10169594	0.009	0.805	0.002	1.002	(0.985, 1.020)			
	rs10182416	0.007	0.241	0.008	1.009	(0.994, 1.023)			
	rs1048932	0.006	0.759	−0.002	0.998	(0.986, 1.011)			
	rs1064213	0.006	0.300	−0.007	0.993	(0.981,1.006)			
ukb-b-19953	rs1000096	0.007	0.638	0.007	1.004	(0.988,1.018)	0.277	0.001	0.371
	rs10099330	0.008	0.494	0.008	0.995	(0.980, 1.010)			
	rs10144067	0.005	0.599	0.005	1.003	(0.992,1.013)			
	rs10169594	0.008	0.805	0.008	1.002	(0.986,1.018)			
	rs10182416	0.007	0.241	0.007	1.009	(0.994,1.024)			
	rs1064213	0.006	0.300	0.006	0.993	(0.981, 1.006)			

In the sensitivity analysis, the MR-egger result indicated that there was no horizontal pleiotropy among the six instrumental variables (*p* > 0.05). The results of Cochran’s Q test were all *p* > 0.05, indicating that there was no significant heterogeneity among the instrumental variables. The MR-PRESSO result showed no horizontal pleiotropy and possible violations of causal effects in the IVs (*p* > 0.05, Table 2). Using the leave-one-out method, the results obtained after removing each SNP one by one were all *p* < 0.05, consistent with the results of the causal effect analysis using the IVW method, indicating that there were no non-specific SNPs affecting the causal estimation results.

## Discussion

This study employed the MR methodology to analyze the potential causal association between BMI and AF using genome-wide association study (GWAS) data. The results showed that all six groups of IVs associated with BMI consistently indicated a significant role of obesity in the pathogenesis of AF.

Previous observational studies have explored the correlation between BMI and the incidence of AF. Bayrak et al. (Bayrak and Altintas, 2022) found a positive association between obesity and recurrence after AF surgery. Lu et al. (Lu et al., 2019) conducted a retrospective study of 790 patients with AF and observed a positive correlation between high BMI and both the high incidence and recurrence rate of AF. Similarly, Schwandner et al. (Schwandner, 2011) observed in a prospective cohort study that the recurrence rate of AF was significantly lower in non-obese patients compared to obese patients. Additionally, the reoperation rate for recurrent abscesses requiring drainage was significantly higher in obese patients. These findings are consistent with the results of our study (OR = 1.002, 95% CI 1.000–1.004). The main symptom of AF is periodic purulent discharge and pain, which can be cured by surgical removal of the infected focus and epithelialized fistula. Obesity or overweight is a major risk factor for the development of comorbid conditions such as cardiovascular disease, type 2 diabetes mellitus, malignancy, asthma, osteoarthritis, chronic back pain, obstructive sleep apnea, non-alcoholic fatty liver disease, and gallbladder diseases (Mulita et al., 2021). Additionally, adipose tissue has high metabolic activity and can secrete pro-inflammatory cytokines such as interleukin (IL)-6 and tumor necrosis factor (TNF)-α, which may promote inflammatory responses and tumorigenesis (Wei et al., 2015; Ying et al., 2020). Physical exercise not only effectively reduces body fat but also improves cardiopulmonary function. Unick et al. (Unick et al., 2017) found that 150–250 min of physical activity per week is sufficient to maintain weight loss. Adults and older adults should engage in at least 150 min per week of moderate-to-vigorous physical activity to prevent significant weight gain and reduce the risk of chronic diseases, while also incorporating muscle-strengthening activities on at least 2 days per week (World Health Organization, 2020).

Despite numerous clinical studies indicating a correlation between BMI and AF, these findings may only reflect the clinical signs of AF patients and do not necessarily establish a causal link between BMI and AF. As previously mentioned, RCTs may be influenced by confounding factors and reverse causality. In contrast to previous observational studies, this study is the first to systematically explore the potential causal association between obesity and AF pathogenesis from a genetic perspective, effectively avoiding the confounding biases and reverse causality issues inherent in traditional observational studies. On one hand, Mendelian randomization studies, leveraging genetic approaches, employed various statistical methods such as IVW, WME, MR-Egger regression analysis, and MR-PRESSO to validate the stability of the causal relationship between exposure factors and outcomes. On the other hand, the data used in this study originated from a European population, ensuring that all study participants were of European ancestry, which minimized biases resulting from differences in environment, ethnicity, and dietary structure. Most importantly, the genetic variants associated with BMI in the six groups were derived from large-scale GWAS meta-analyses, ensuring the strength of IVs in the MR analysis and the accuracy of the study results.

However, this study also has some limitations. Firstly, the inclusion of only European individuals reduces population stratification bias but may limit the reliability of the evidence for the association between BMI and AF in other ethnic groups. Therefore, further studies are needed to investigate the relationship between BMI and AF in other ethnic populations. Secondly, AF exhibits multiple clinical subtypes, necessitating subsequent subgroup studies, including exploring the relationship between normal weight, overweight and obese people on the formation and postoperative healing of AF, complex AF and high AF. Thirdly, this study solely explored the causal relationship between BMI and AF without delving into the underlying mechanisms. Adjusting or maintaining normal BMI may be a potential treatment for alleviating the clinical symptoms of anal fistula and improving the postoperative recovery of anal fistula surgery. Meantime, future studies should be conducted across different ethnic groups and include stratified analyses based on gender and age to further explore the impact of BMI on the occurrence and development of AF. Additionally, BMI changes over time, and while the aim of MR analysis is to estimate the lifelong impact of exposure factors on outcomes, the genetic instruments used here are associated with BMI at specific age intervals, potentially affecting the validity of the results. Although Mendelian randomization is a commonly used experimental design, it also has its limitations. In practical applications, it is crucial to consider multiple factors comprehensively and in light of specific circumstances to ensure the reliability of experimental results. Given that AF can also result from multiple factors, including rectovaginal fistulas caused by perineal colostomy, it is crucial to be mindful of this complication. Additionally, since the GWAS database does not track the formation of AF, it is important to consider possible exposures (Mulita et al., 2022).

In summary, this study employed a two-sample MR approach to systematically investigate the potential causal association between BMI and the occurrence of AF. The results indicate a positive correlation between BMI and the risk of AF, suggesting that correcting obesity may have a preventive role in the occurrence and development of AF. However, further multicenter clinical studies with larger sample sizes and additional MR studies are needed to validate the causal relationship between obesity and AF pathogenesis.

## Data Availability

The original contributions presented in the study are included in the article/Supplementary Material, further inquiries can be directed to the corresponding author.
